# Focused ultrasound-mediated temozolomide delivery into intact blood–brain barrier tissue improves survival in patient-derived xenograft model of glioblastoma

**DOI:** 10.1186/s12987-025-00695-0

**Published:** 2025-08-25

**Authors:** Jaewoo Shin, Jin-Kyoung Shim, Chanho Kong, Younghee Seo, Sangheon Han, Seok-Gu Kang, Won Seok Chang

**Affiliations:** 1 Medical Device Development Center, Daegu-Gyeongbuk Medical Innovation Foundation (K-MEDI Hub), Daegu, 41061 Republic of Korea; 2 Department of Neurosurgery, Stereotactic and Functional Neurosurgery Division, Severance Hospital, Yonsei University College of Medicine, 50 Yonsei-ro, Seodaemun-gu, Seoul, 03722 Republic of Korea; 3 Brain Research Institute, Yonsei University College of Medicine, Seoul, 03722 Republic of Korea; 4 Department of Neurosurgery, Brain Tumor Center, Severance Hospital, Yonsei University College of Medicine, Seoul, 03722 Republic of Korea; 5 Brain Tumor Translational Research Laboratory, Department of Biomedical Sciences, Yonsei University College of Medicine, Seoul, 03722 Republic of Korea; 6 Bionics Research Center, Biomedical Research Division, Korea Institute of Science and Technology, Seoul, 02792 Republic of Korea; 7 Department of Medical Science, Yonsei University Graduate School, Seoul, 03722 Republic of Korea

**Keywords:** Blood–brain barrier, Focused ultrasound, Glioblastoma, Temozolomide, Tumorsphere

## Abstract

**Background:**

Glioblastoma (GBM) is the most prevalent and aggressive primary brain tumor in adults, characterized by rapid proliferation and invasive infiltration into normal brain tissue. Despite maximal resection and temozolomide (TMZ) chemotherapy, over 80% of GBM cases recur near the resection margin, highlighting the need for improved therapeutic strategies. The blood–brain barrier (BBB) remains a major obstacle to effective drug delivery, limiting TMZ penetration into infiltrative tumor regions. This study explores the potential of focused ultrasound (FUS) to transiently open the BBB, optimizing TMZ delivery to GBM-infiltrated brain regions before tumor neovascularization, and investigates its impact on tumor progression and survival in an orthotopic xenograft mouse model.

**Methods:**

Human primary GBM tumorspheres (TS15-88) were implanted into the striatum of 4- to 8-week-old male athymic nude mice to establish an orthotopic xenograft model. FUS was applied 1 week post-implantation, followed by intraperitoneal TMZ administration. BBB permeability was assessed using Evans blue extravasation, gadolinium-enhanced T1-weighted magnetic resonance imaging (MRI), and ZO-1 tight junction protein expression. GBM infiltration into the brain was confirmed using ZEB-1 and hematoxylin and eosin staining. Bioluminescence imaging and Kaplan–Meier survival analysis were used to evaluate the therapeutic effects of combined FUS and TMZ treatment.

**Results:**

MRI and Evans blue staining confirmed that BBB integrity was preserved in the tumor-only group, suggesting that tumor-induced neovascularization had not yet developed at the time of treatment. However, FUS-mediated BBB opening significantly enhanced Evans blue extravasation and reduced ZO-1 expression, indicating transient and localized BBB disruption. FUS-TMZ combination therapy significantly suppressed tumor growth, as evidenced by bioluminescence imaging, and prolonged survival compared to that with TMZ alone. Additionally, applying FUS in the early treatment phase (1-day group) showed a trend toward better tumor suppression and survival outcomes compared to that at later time points.

**Conclusions:**

Our findings suggest that integrating FUS with standard TMZ chemotherapy during the early treatment phase may enhance drug penetration into infiltrative tumor regions, leading to improved tumor control and survival outcomes. These results highlight the clinical potential of FUS as an adjunct therapy to optimize TMZ efficacy, particularly in patients with early-stage GBM.

**Graphical abstract:**

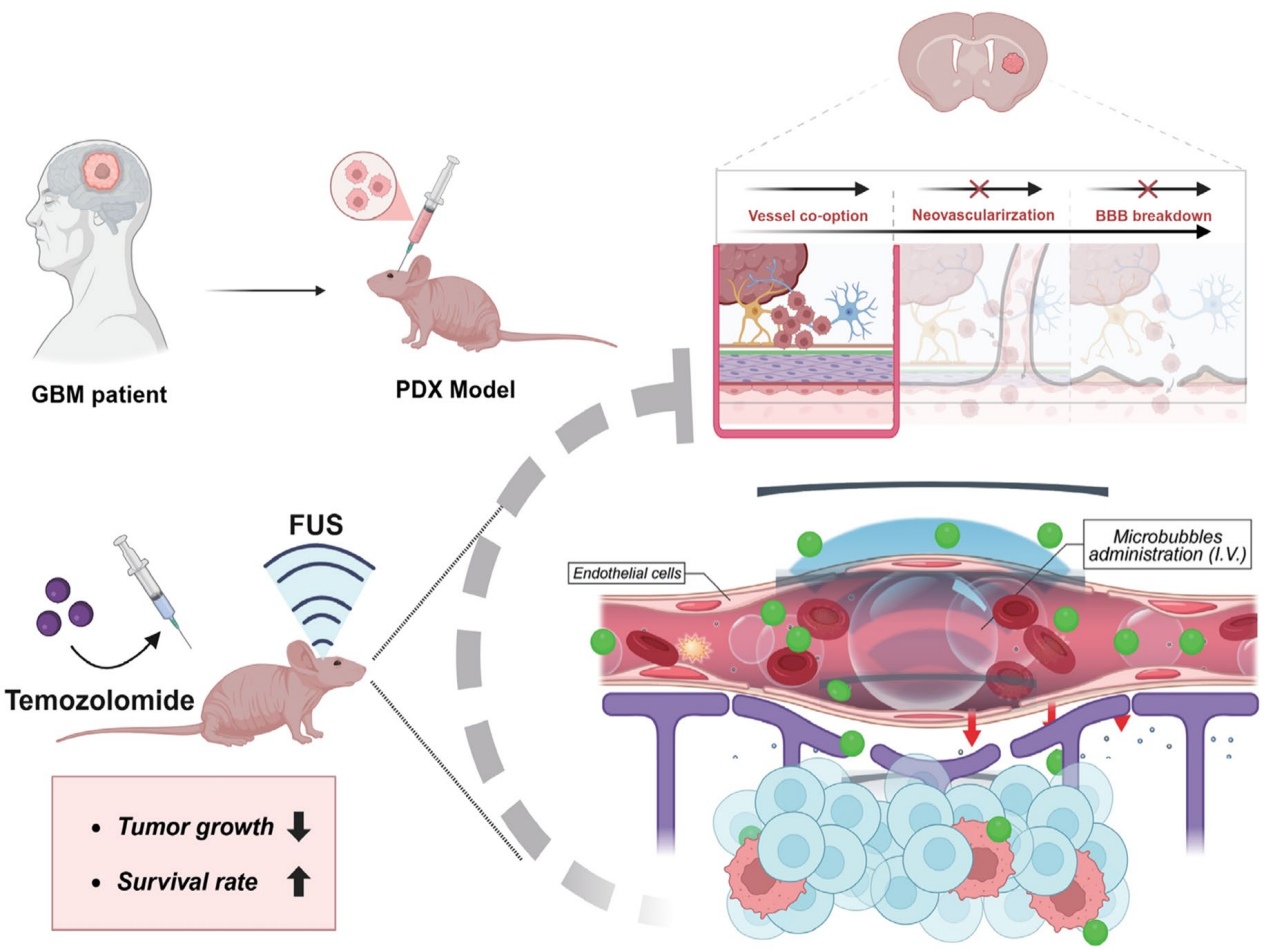

**Supplementary Information:**

The online version contains supplementary material available at 10.1186/s12987-025-00695-0.

## Background

Glioblastoma (GBM) is the most common and aggressive type of primary brain tumor in adults, characterized by its high proliferation rate and invasive tendencies into normal brain parenchyma [1]. Despite the current standard of care, including maximal tumor resection followed by temozolomide (TMZ) chemotherapy, over 80% of patients with GBM experience recurrence near the surgical resection site [2, 3]. However, effective therapeutic strategies remain largely unknown [4, 5]. Although TMZ is the first-line chemotherapy for GBM, its efficacy is significantly limited by drug resistance and restricted permeability across the blood–brain barrier (BBB) [6, 7]. The BBB and blood–tumor barrier (BTB) act as major obstacles, limiting the penetration of therapeutic agents into infiltrative tumor regions [8]. Furthermore, current imaging techniques, including computed tomography and gadolinium-enhanced T1-weighted magnetic resonance imaging (MRI), often fail to detect microscopic invasion beyond the visible tumor margin [9]. This limitation commonly results in incomplete tumor removal, increased recurrence risk, or excessive resection of healthy brain tissue, leading to neurological deficits [10, 11].

One promising approach to temporarily and noninvasively enhance BBB permeability is the use of focused ultrasound (FUS) [12, 13]. When combined with intravenously administered microbubbles, FUS generates transient and localized BBB disruption, enabling enhanced drug delivery to the brain [14, 15]. Previous studies have demonstrated the efficacy of FUS-mediated drug delivery in various neurological disorders, including Alzheimer’s disease, Parkinson’s disease, and traumatic brain injury [16–21]. Moreover, FUS modulates the brain microenvironment by influencing immune responses and inflammatory signaling [22, 23].

Although FUS has been widely studied for GBM drug delivery, most prior research has focused tumors with established neovascularization [24, 25]. It remains unclear whether FUS-mediated BBB opening (BBBO) at an earlier stage, before tumor neovascularization, can improve therapeutic outcomes. Pre-vascularized tumor regions often retain an intact BBB, presenting a critical window to optimize TMZ delivery before abnormal vascularization complicates drug penetration.

In this study, we employed an orthotopic xenograft mouse model implanted with patient-derived GBM tumorspheres (TS15-88), a relevant platform [26, 27] for assessing drug efficacy in GBM cells [28], to investigate whether FUS-mediated BBBO enhances TMZ delivery in a pre-vascularized GBM environment. We hypothesized that the application of FUS at an early stage would enhance drug penetration, delay tumor progression, and improve survival outcomes. To test this, we assessed BBB permeability, tumor progression, and survival following FUS and TMZ treatment in our xenograft model.

## Materials and methods

### Animals

All experimental procedures with animals were conducted in compliance with the Guide for the Care and Use of Laboratory Animals of the National Institutes of Health and approved by the Institutional Animal Care and Use Committee (IACUC; 2020 − 0248) of Yonsei University. Male athymic nude mice (4–8 weeks old; Central Lab. Animal Inc., Seoul, Korea) were housed in micro-isolator cages under sterile conditions and monitored for at least one week prior to study initiation. Lighting, temperature, and humidity were centrally controlled. The mice were observed daily for signs of stress or illness and acclimatized to handling to minimize experimental variability.

### GBM tumorsphere culture

Primary tumor cells derived from a patient with GBM, TS15-88, were used to establish tumorsphere (TS) models. TS15-88 was established from fresh GBM tissue specimens with the approval of the Institutional Review Board of Yonsei University College of Medicine (IRB No. 4-2021-1319). Written informed consent was obtained from the patient. Cells were cultured in complete TS media composed of Dulbecco’s modified Eagle medium/nutrient mixture F-12 (Mediatech, Manassas, VA, USA), 1× B27 (Invitrogen, San Diego, CA, USA), 20 ng/mL basic fibroblast growth factor (Novoprotein, Summit, NJ, USA), and 20 ng/mL epidermal growth factor (Novoprotein) [27, 29, 30]. The GBM cell line U87 was also cultured under these conditions.

### Characterization of GBM TSs

TS formation from human GBM specimens was performed as previously described [31]. The expression of the stemness markers CD133 and nestin (Abcam, Cambridge, UK) was analyzed using immunocytochemistry. Neuroglial differentiation in TS15-88 cells was evaluated by monitoring the expression of glial fibrillary acidic protein (GFAP; Dako, Carpinteria, CA, USA), myelin basic protein (MBP), neuronal nuclei (NeuN), and tubulin beta 3 (TUBB3; Chemicon, Temecula, CA, USA).

### Cell viability assay

Cell viability after TMZ treatment was assessed using the WST-1 assay (EZ-Cytox; DoGenBio, Korea). Cells (1 × 10⁴ cells/well) were seeded into 96-well plates and incubated at 37 °C for 24 h, followed by treatment with TMZ for 3 days. WST-1 reagent (10 µL/well) was added, and the cells were incubated for an additional 1 h. Absorbance at 450 nm was measured using a microplate reader. The experiments were performed in triplicate, and cell viability was expressed as a percentage of the control cells.

### Orthotopic xenograft mouse model

Mice were anesthetized with Zoletil (30 mg/kg; Virbac Korea, Seoul, Korea) and xylazine (10 mg/kg; Bayer Korea, Seoul, Korea), administered intraperitoneally. Dissociated TS15-88 cells (5 × 10⁵) were implanted into the right frontal lobe at a depth of 4.5 mm using a Hamilton syringe (Dongwoo Science Co., Seoul, Korea) and a guide-screw system [27, 32]. Mice were euthanized in compliance with the approved protocol when their daily monitored body weight decreased by more than 15% relative to the initial weight.

### FUS

FUS was generated using a spherical 0.515-MHz transducer (H-107MR; Sonic Concept Inc., Bothell, WA, USA; diameter, 51.7 mm; curvature radius, 63.2 mm). A waveform generator (33220 A; Agilent, Palo Alto, CA, USA) and a 40-dB RF power amplifier (210 L; ENI Inc., Rochester, NY, USA) were used to drive the transducer [33]. The mice were anesthetized with ketamine (75 mg/kg) and xylazine (4 mg/kg) and positioned in a stereotaxic frame (Narishige, Tokyo, Japan). Definity microbubbles (0.04 mL/kg; Lantheus Medical Imaging, North Billerica, MA, USA) were injected intravenously 10 s prior to sonication.

**Fig. 1:**
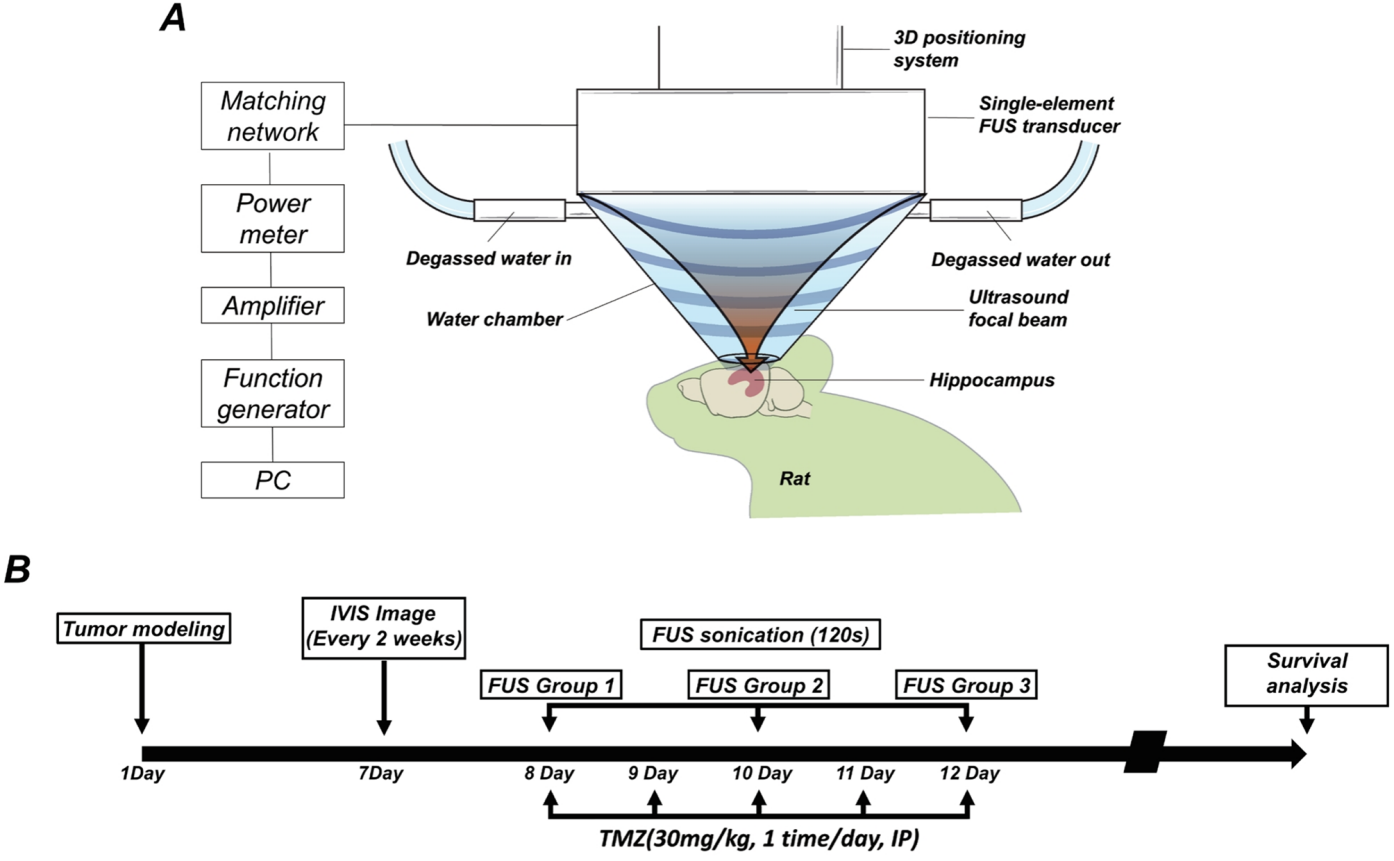
Schematic representation of the FUS experimental setup and timeline of procedures. (**A**) Illustration of the FUS system, including the single-element FUS transducer, degassed water chamber, and 3D positioning system. The transducer is coupled to the target region of a mouse brain via degassed water, ensuring precise delivery of ultrasound energy. Key components of the system include a function generator, amplifier, and power meter, which are used to control and monitor the ultrasound output. (**B**) Timeline of the experimental protocol for TMZ administration and FUS application. TMZ was injected intraperitoneally once daily for 5 consecutive days, starting on the eighth day post-xenograft implantation. FUS treatments were conducted on the first, third, and fifth days during the TMZ administration period. Bioluminescence imaging was performed at 2-week intervals to monitor tumor progression and treatment efficacy

The FUS parameters included 10-ms bursts, a 1-Hz repetition rate, and a peak negative pressure of 0.25 MPa for 2 min. FUS was precisely targeted to the tumor implantation site using MRI-guided stereotactic coordinates and performed on the first, third, and fifth days during TMZ treatment. TMZ (30 mg/kg) was administered intraperitoneally daily for five consecutive days, with injections performed immediately after each FUS session. Bioluminescence imaging was performed at 2-week intervals, starting 1 week after xenograft implantation. Figure 1 illustrates the experimental setup for FUS and the timeline of TMZ administration.

### MRI

MRI was performed using a 9.4-T Bruker system (Biospec 94/20 USR; Bruker, Ettlingen, Germany) with a rat head coil. BBBO was confirmed using gadolinium-enhanced T1-weighted imaging. T2-weighted images were used to detect edema and tissue damage. Gadolinium contrast agent (0.2 mL/kg; Gadovist, Bayer Schering Pharma AG, Berlin, Germany) was injected intravenously immediately after each FUS session for MRI confirmation of BBBO. The MRI sequences used are summarized in Table 1.

Percent enhancement was calculated by measuring the average pixel intensity within a 2-mm × 2-mm voxel region of interest at each targeted spot, comparing it to an untreated reference region, and then averaging these values across all focal spots within each animal (Image J; NIH, Bethesda, MD, USA).

**Table 1:** Microscopy parameters

	T1-weighted imaging	T2- weighted imaging
FOV (cm)	3.5	3.5
TR (ms)	350	2500
TE (ms)	5.4	33
Matrix	256 × 256	256 × 256
FA (deg)	40	180
SL (mm)	1	1

FOV field of view, TR repetition time, TE time to echo, FA fractional anisotropy, SL Slice thickness.

### BBB permeability assay

Evans blue dye (2% in saline, 100 mg/kg; Sigma-Aldrich, MO, USA) and gadolinium contrast agent were administered intravenously immediately after FUS sonication. After 4 h of circulation, brains were harvested, weighed, homogenized, and processed using trichloroacetic acid. The extravasation of Evans blue dye was quantified using a spectrophotometer at 620 nm [34].

### Histological analysis

Brain tissues were fixed via transcardial perfusion with 0.9% saline followed by 4% paraformaldehyde. Paraffin-embedded brains were sectioned (6 μm) and stained with hematoxylin and eosin (H&E; H-3401, Vector Laboratories, CA, USA) for pathological examination. For immunostaining, sections were incubated with a human-specific primary antibody against ZEB1 (1:200; Abcam), followed by detection with an Alexa Fluor 488-conjugated secondary antibody. Nuclei were counterstained with DAPI (1 µg/mL; Sigma-Aldrich). Images were captured using a Zeiss LSM 710 confocal microscope (Carl Zeiss, Oberkochen, Germany).

### Western blot analysis

One hour after sonication, the mice (*n* = 15 per group) were anesthetized with ketamine (75 mg/kg) and xylazine (4 mg/kg). The right frontal lobe was dissected, and 1 mm coronal brain slices were prepared using a brain slicer. The tissues were homogenized in protein extraction solution containing 1.0 mM EDTA, 1.0 mM PMSF, 1 µM pepstatin, 1 µM aprotinin, and 1 µM leupeptin (PRO-PREP, iNtRON Biotechnology, Seongnam, Korea). Protein concentrations were determined using a BCA kit (Pierce, Rockford, IL, USA).

Proteins (20 µg per sample) were separated via 10% sodium dodecyl sulfate-polyacrylamide gel electrophoresis at 100 V for 1 h and transferred onto polyvinylidene fluoride membranes at 100 V for 90 min. Membranes were blocked in 5% non-fat dry milk dissolved in phosphate-buffered saline-Tween 20 (0.05% Tween 20) for 1 h at room temperature.

Primary antibodies, rabbit polyclonal anti-ZO-1 (1:1000; Thermo Fisher Scientific, Waltham, MA, USA) and mouse monoclonal anti-β-actin (1:20,000; Sigma-Aldrich), were incubated overnight at 4 °C. Secondary antibodies, goat anti-rabbit IgG(H + L)-HRP (1:2000; GenDEPOT, Baker, TX, USA) and goat anti-mouse IgG(H + L)-HRP (1:20,000; GenDEPOT), were applied for 2 h at room temperature.

Protein bands were visualized using enhanced chemiluminescence (WEST-Queen Western Blot Detection Kit; iNtRON Biotechnology) and quantified using an LAS 4000 Mini imaging system (GE Healthcare Life Sciences, Marlborough, MA, USA). Band intensities were normalized to β-actin as the loading control and analyzed using Multi Gauge software (version 3.0; Fujifilm, Tokyo, Japan).

### Bioluminescence imaging

Bioluminescence acquisition and analyses were performed using an In Vivo Imaging System (Caliper Life Sciences, Hopkinton, MA, USA) and Living Image v4.2 software (Revvity, Waltham, MA, USA). Mice were injected intraperitoneally with 100 µL of D-luciferin (30 mg/mL; Promega, Madison, WI, USA) 15 min prior to imaging to allow substrate metabolism and achieve peak bioluminescence. Imaging was conducted under 2.5% isoflurane anesthesia, with exposure times of 5 s and a medium field of view.

Bioluminescence signals were quantified as total flux (photons/s) within manually defined regions of interest using Living Image software. Background signals were subtracted, and the data were normalized to the baseline measurements taken prior to the treatment. Total flux was additionally measured at week 9 post-implantation to assess tumor growth. Grayscale photographic images and bioluminescence color maps were superimposed for visual representation of the results.

### Quantification of TMZ Delivery via LC-MS/MS

To assess whether FUS-mediated BBBO enhanced the delivery and metabolic outcome of TMZ in the target brain region, liquid chromatography-mass spectrometry (LC-MS/MS) analysis was performed. The study involved a Control group (no treatment, *n* = 3), a TMZ-only group (*n* = 4), and a TMZ + FUS group (*n* = 4). On day 7 post-xenograft implantation, TMZ (30 mg/kg) was administered intraperitoneally in the TMZ-only and TMZ + FUS groups. For the TMZ + FUS group, TMZ was injected immediately after FUS. Brain tissues were harvested precisely 4 h after TMZ injection, and samples from the sonicated tumor-bearing hemisphere were collected for analysis. For metabolite extraction, 500 µL of methanol was added to each tissue sample, followed by vortexing for 1 min and sonication for 20 min. The resulting homogenates were subjected to centrifugation at 3,000 RPM for 3 min to precipitate proteins and cellular debris. The cleared supernatants were then collected and filtered through a 0.45-µm syringe filter prior to analysis.

The analysis was conducted at the Yonsei University Core Research Facility using an LC-MS/MS system equipped with a Heated Electrospray Ionization (H-ESI) source. Chromatographic separation was achieved on a Hypersil Gold C18 column maintained at 40 °C. For each sample, a 5-µL aliquot was injected, and metabolites were separated at a flow rate of 0.3 mL/min. The mobile phases consisted of 0.1% formic acid in water (Solvent A) and 0.1% formic acid in methanol (Solvent B), applied with a gradient elution profile.

Mass spectrometric data were acquired in both positive and negative ion modes over a scan range of m/z 70–1000. Full scan MS1 data was collected at a resolution of 120,000, while data-dependent MS/MS (ddMS²) scans were acquired at a resolution of 15,000 with a normalized HCD collision energy of 30%.

Given the rapid in vivo hydrolysis of TMZ, the relative quantification of drug delivery and efficacy was based on the peak area of its stable, terminal metabolite, 5-aminoimidazole-4-carboxamide (AIC), corresponding to the [M + H]⁺ ion at m/z 127.0614. The relative abundance of AIC was compared across the Control, TMZ-only, and TMZ + FUS groups to determine the effect of each treatment condition.

### Statistical analysis

Tukey’s post-hoc comparisons were used in conjunction with one-way analysis of variance (ANOVA) to examine the data, and survival analysis was performed using Kaplan–Meier curves and the log-rank test. GraphPad Prism 10 (GraphPad Software, Boston, MA, USA) was used for quantitative analysis. The mean ± standard error of the mean is used to show the data. Statistical significance was set at *p < 0.05, **p < 0.01, and ***p < 0.001.

## Results

### Characterization of GBM TSs (TS15-88)

**Fig. 2:**
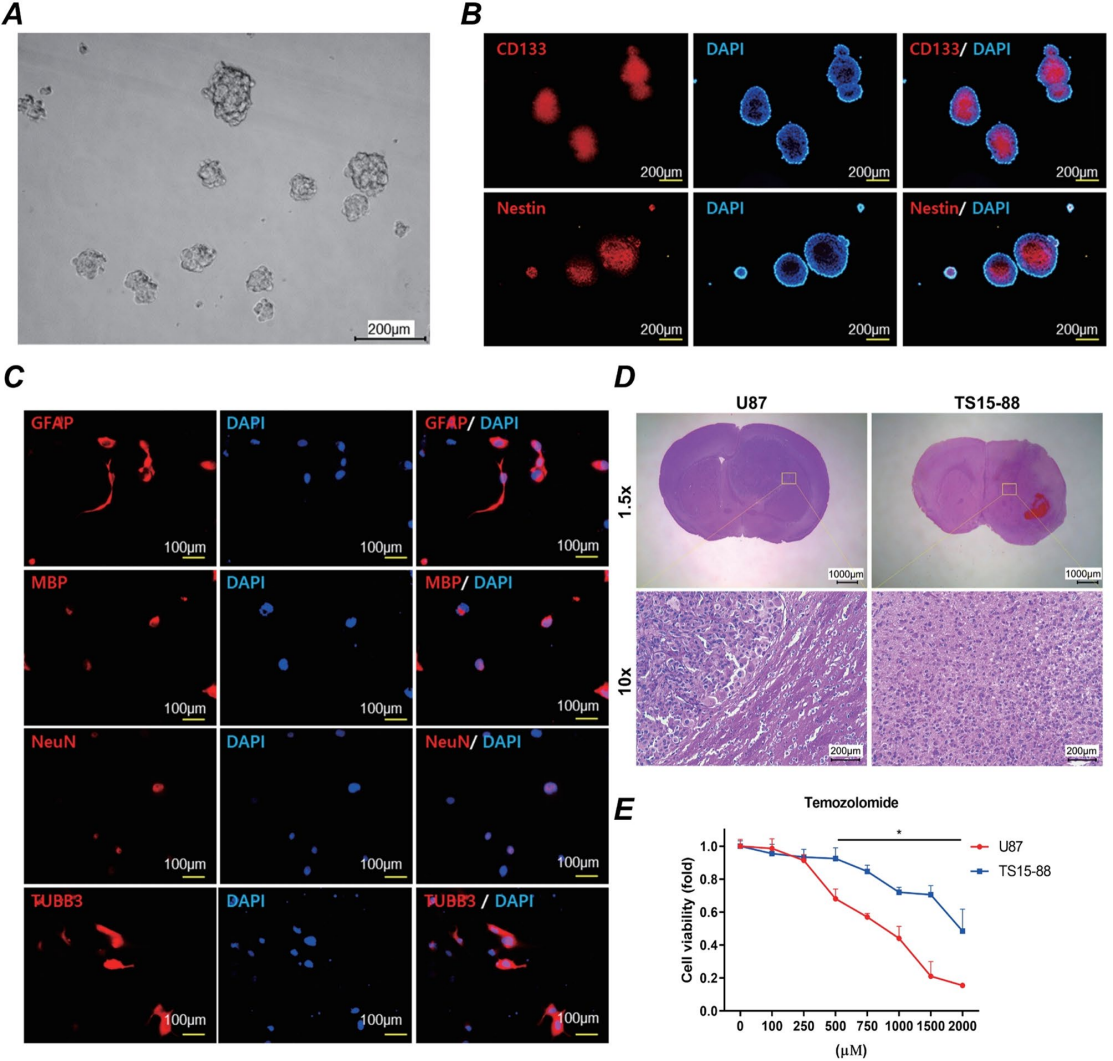
Characterization of TS15-88 cells and their response to TMZ. (**A**) Representative brightfield image showing the morphology of TS15-88 TSs (scale bar = 200 μm). (**B**) Expression of stemness markers CD133 and nestin in TS15-88 cells, visualized using immunocytochemistry (red). Nuclei are counterstained with DAPI (blue). Merged images confirm co-localization of the markers with nuclear regions (scale bar = 200 μm). (**C**) Neuroglial differentiation potential of TS15-88 cells evaluated by immunocytochemistry for GFAP, MBP, NeuN, and TUBB3 (red). Nuclei are counterstained with DAPI (blue). Merged images show the differentiation capacity into both glial and neuronal lineages (scale bar = 100 μm). (**D**) Hematoxylin and eosin (H&E) staining of brain sections obtained from mice injected with either TS15-88 or U87 cells. (**E**) Cell viability assay results for TS15-88 and U87 cells treated with various concentrations of TMZ. Cell viability assessed 72 h after TMZ treatment: TS15-88 cells exhibited greater resistance to TMZ than U87 cells, as indicated by their higher viability at equivalent TMZ concentrations. Data are presented as mean ± standard error of the mean (SEM) (*n* = 8 per group)

The TS15-88 TSs, derived from a patient with GBM, exhibited a compact spherical morphology under brightfield microscopy (Fig. 2A). Immunocytochemical analysis confirmed the expression of stemness markers CD133 and nestin, indicating their stem-like properties (Fig. 2B). Co-localization with DAPI-stained nuclei further validated these findings. To assess the neuroglial differentiation potential of TS15-88 cells, immunocytochemistry was performed for glial (GFAP, MBP) and neuronal (NeuN, TUBB3) markers. TS15-88 cells were able to differentiate into both glial and neuronal lineages, as evidenced by positive staining for these markers (Fig. 2C). Histological analysis showed that TS15-88 tumors exhibited a more diffuse and infiltrative growth pattern compared with the localized growth of U87 tumors (Fig. 2D). Cell viability assays were conducted 72 h after TMZ treatment. TS15-88 cells exhibited higher resistance to TMZ than U87 cells, maintaining greater viability across a range of TMZ concentrations (Fig. 2E). These findings demonstrated that TS15-88 cells closely mimic the chemoresistant phenotype observed in patient-derived GBM, making them a suitable model for further studies.

### Confirmation of orthotopic xenograft model

**Fig. 3:**
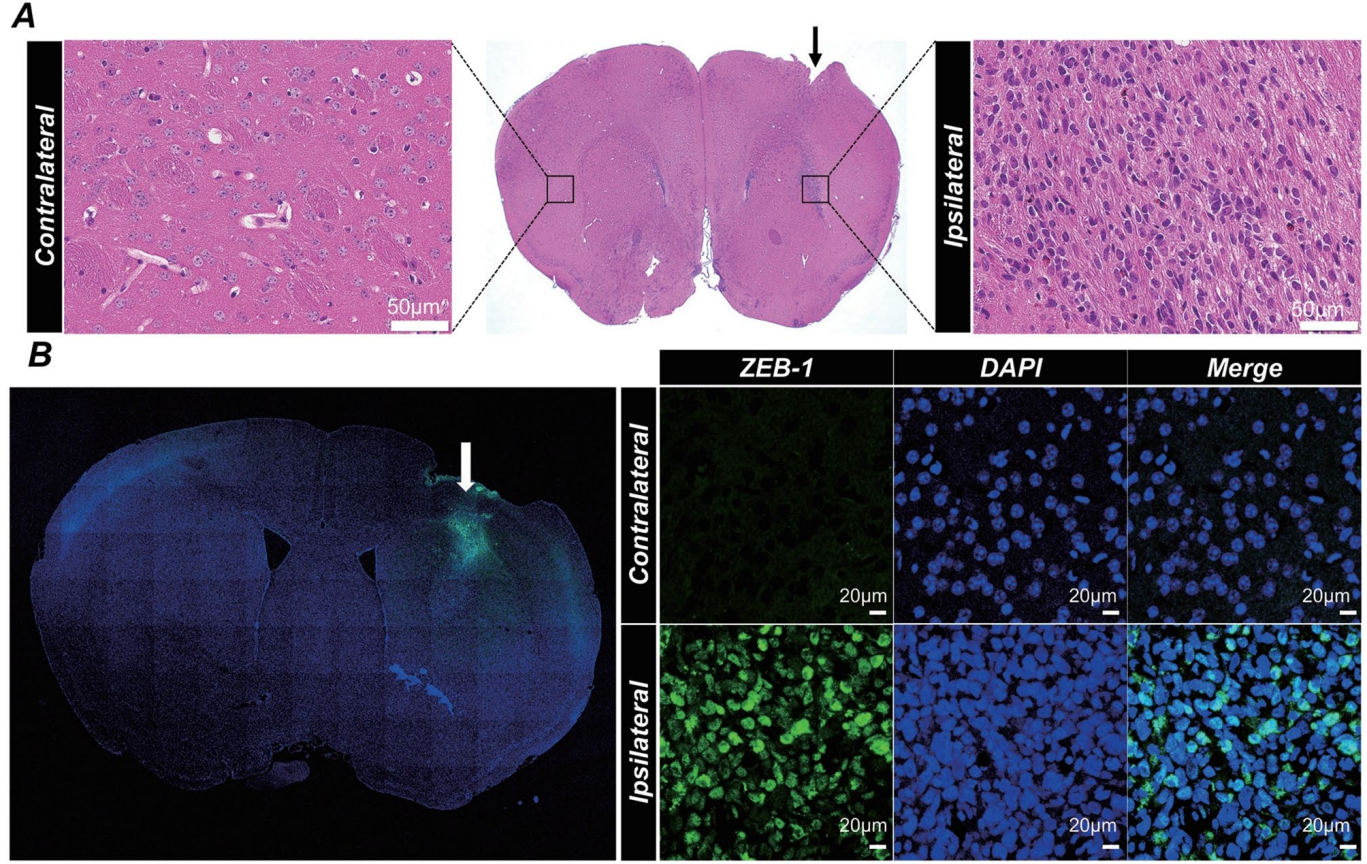
Confirmation of orthotopic xenograft mouse model. (**A**) Representative H&E staining of contralateral and ipsilateral brain hemispheres 7 days after implantation of patient-derived GBM cells (TS15-88). The middle panel shows a coronal section with tumor infiltration localized to the ipsilateral hemisphere. The arrowhead indicates the site of tumor cell injection (scale bar = 50 μm for contralateral and ipsilateral close-up images). (**B**) Immunofluorescence staining for ZEB-1 (green) in contralateral and ipsilateral brain hemispheres using a human-specific antibody to confirm the presence of patient-derived GBM cells. Nuclei are counterstained with DAPI (blue). Merged and orthogonal views reveal the localization of ZEB-1-positive cells in the ipsilateral hemisphere, confirming tumor cell engraftment 7 days post-implantation (scale bar = 20 μm)

The tumorigenic potential of TS15-88 cells was evaluated using an orthotopic xenograft mouse model. Seven days post-implantation, histological analysis revealed distinct tumor formation in the ipsilateral hemisphere, whereas the contralateral hemisphere remained unaffected. H&E staining revealed dense tumor cell clusters localized to the implantation site (Fig. 3A).

Immunofluorescence staining with a human-specific ZEB-1 antibody confirmed the presence of TS15-88 cells exclusively in the ipsilateral hemisphere. Co-localization with DAPI-stained nuclei further validated the identity of these tumor cells (Fig. 3B). Orthogonal views provided additional confirmation of their spatial integration within the brain tissue.

These results demonstrated the successful engraftment of TS15-88 cells in the orthotopic xenograft model, replicating key features of patient-derived GBM, including localized infiltration and robust tumor formation.

### FUS enhances BBB permeability in the orthotopic xenograft model

**Fig. 4:**
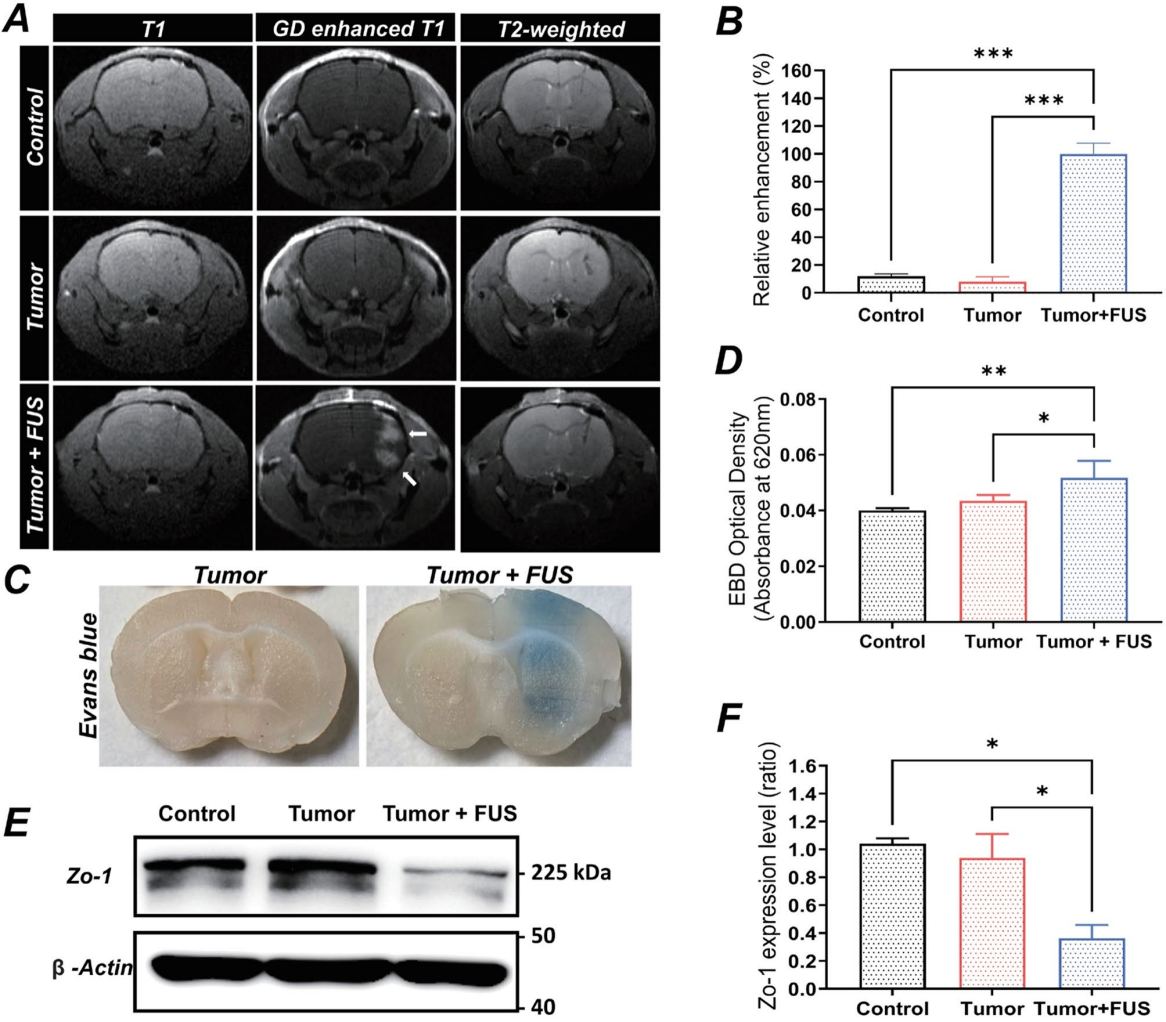
Evaluation of BBB permeability and tight junction integrity in orthotopic xenograft model following FUS treatment. (**A**) Representative T1-weighted, gadolinium-enhanced T1-weighted, and T2-weighted MRI images captured immediately after FUS treatment in the control, tumor-only, and tumor + FUS groups. The tumor + FUS group shows localized gadolinium enhancement in the sonicated region, indicating successful BBB opening. (B) Quantitative analysis of relative enhancement derived from T1-gadolinium-enhanced MRI images immediately following FUS treatment Data are presented as mean ± SEM (*n* = 3 per group). (**C**) Representative macroscopic images demonstrating Evans blue dye extravasation in tumor and tumor + FUS groups, 4 h post-FUS treatment. The tumor + FUS group shows Evans blue dye distribution. (**D**) Quantification of Evans blue extravasation 4 h after FUS treatment. The tumor + FUS group exhibits significantly greater dye levels than the control and tumor-only groups. Data are presented as mean ± SEM (*n* = 3 per group). (E) Western blot analysis of ZO-1 expression in the control, tumor-only, and tumor + FUS groups 4 h after FUS treatment. The tumor + FUS group displays reduced ZO-1 levels, indicating tight junction disruption. (F) Quantification of ZO-1 western blot band intensities. Data are presented as mean ± SEM (*n* = 5 per group). Statistical significance: *p < 0.05, **p < 0.01

One of the major challenges in GBM treatment is overcoming the BBB to improve drug delivery to the tumor. MRI experiments demonstrated localized contrast enhancement on T1-weighted gadolinium-enhanced images immediately following sonication, confirming successful BBB opening (Fig. 4A). Quantitative analysis of relative enhancement further confirmed significant increases in BBB permeability after FUS treatment compared with the Control and tumor-only groups (Fig. 4B). The BBB permeability assay performed 4 h post-FUS further confirmed BBBO, showing increased Evans blue extravasation specifically at the FUS-targeted region (Fig. 4C, D). Additionally, Western blot analysis indicated significant downregulation of the tight junction protein ZO-1 in the FUS-treated group compared with controls, indicating transient disruption of tight junction integrity (Fig. 4E, F).

Notably, in the tumor-only group, no significant changes were observed in MRI contrast enhancement, Evans blue dye extravasation, or ZO-1 expression, indicating that tumor-induced neovascularization had not yet developed at this stage (Fig. 4A–F). These results suggest that the BBB remained largely intact in the early-stage tumor environment, making this a suitable model for evaluating the impact of FUS on BBB permeability before pathological vascularization occurs.

### Evaluation of FUS timing in BBBO

To determine the optimal timing of FUS administration, FUS was applied on the first, third, and fifth days during TMZ treatment, and tumor progression was monitored using bioluminescence imaging and survival analysis. As shown in Supplementary Fig. 1, no statistically significant differences were observed between the three groups. However, the 1-day FUS group exhibited a trend toward slower tumor progression and prolonged survival compared with the 3-day and 5-day groups. These results suggest that applying FUS earlier in the treatment regimen may have a slightly better therapeutic effect; however, further studies are needed to confirm this observation.

### FUS enhances tumor responses in the orthotopic xenograft model

**Fig. 5:**
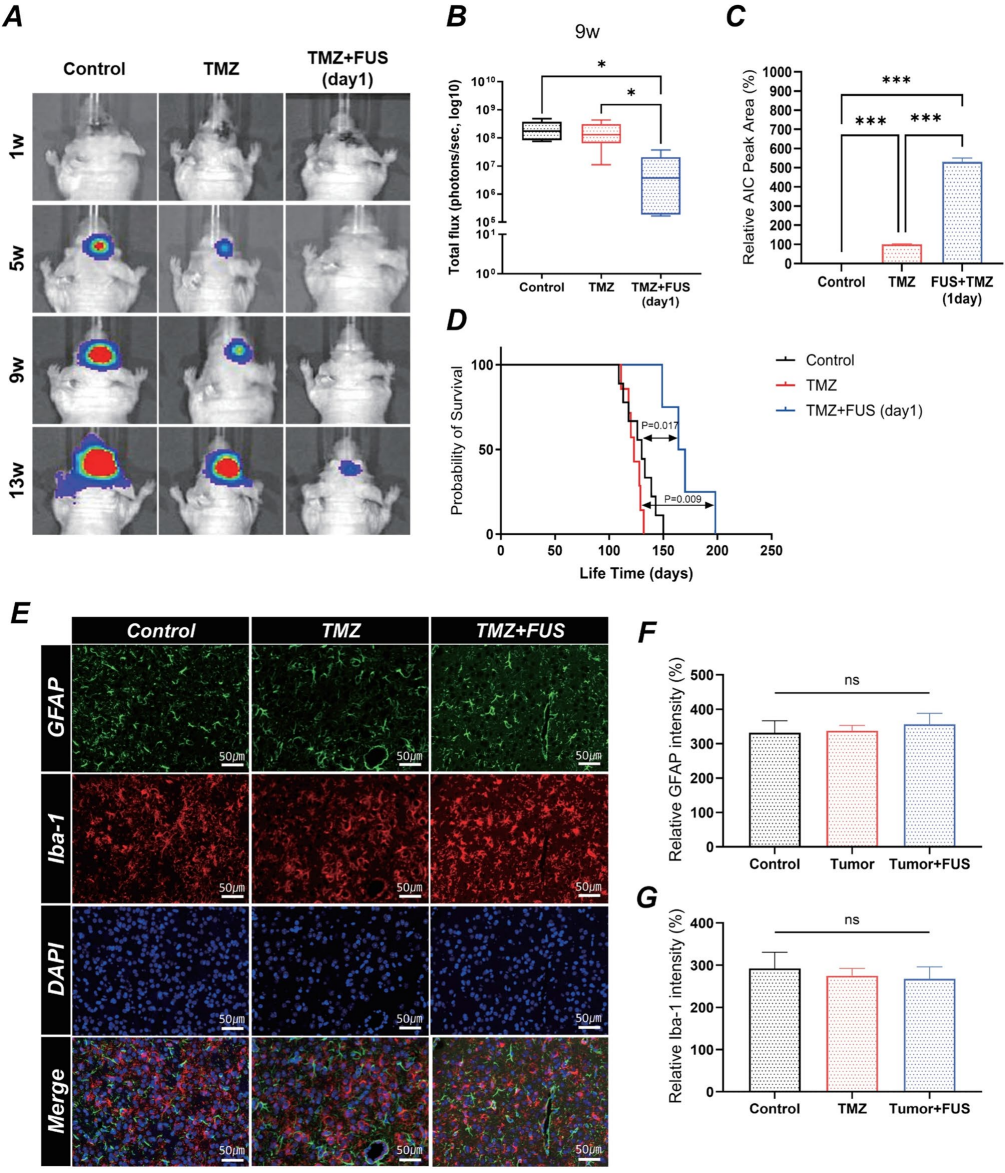
Therapeutic efficacy of TMZ and FUS in an orthotopic xenograft mouse model. (**A**) Representative bioluminescence imaging showing tumor progression in the control, TMZ-only, and TMZ + FUS groups at weeks 1, 5, 9, and 13 post-treatment. Tumor burden is indicated by the intensity of the bioluminescence signal, with a higher intensity representing larger tumors. (**B**) Quantification of tumor volume based on bioluminescence signal intensity (total photon flux) over time. Statistical analysis was performed using one-way ANOVA followed by Tukey’s post-hoc test (*p < 0.05, **p < 0.01). Data are presented as mean ± SEM (*n* = 5 per group). (**C**) LC-MS/MS quantitative analysis of intraparenchymal TMZ concentrations in sonicated (FUS-treated) versus non-sonicated brain tissues. (D) Kaplan–Meier survival analysis of individual mouse groups. Mice treated with TMZ + FUS showed significantly prolonged survival compared to those in the control and TMZ-only groups. Statistical significance: *p < 0.05, **p < 0.01. (E-G) Representative immunofluorescence images showing Iba-1 (green, microglial activation marker), GFAP (red, astrocytic activation marker), and DAPI (blue, nuclear staining) in Control, TMZ, FUS, and TMZ + FUS groups. No significant differences in immune or glial cell activation were observed among the groups. Scale bars: 50 μm

The therapeutic impact of TMZ combined with FUS was evaluated using bioluminescence imaging. At the 9-week follow-up, the TMZ + FUS group showed significantly reduced tumor progression compared to both Control and TMZ-only groups, as demonstrated by quantification of total photon flux (Fig. 5A, B). Additionally, quantitative LC-MS/MS analysis of TMZ concentrations demonstrated significantly increased intratumoral TMZ delivery in the TMZ + FUS group compared with the TMZ-alone group (Fig. 5C). This confirms that FUS-mediated BBBO effectively enhances TMZ penetration, correlating with the observed suppression of tumor progression. Kaplan-Meier survival analyses further supported these findings, demonstrating significantly prolonged survival in the TMZ + FUS-treated group compared with the Control and TMZ-only groups (Fig. 5D).

To evaluate potential immune modulation induced by FUS, immunofluorescence staining for GFAP and Iba-1 was performed (Fig. 5E–G, Supplementary Fig. 2). However, no significant differences in GFAP or Iba-1 expression were observed among the Control, tumor-only, FUS-alone, TMZ-alone, and TMZ + FUS groups. These results suggest that the enhanced therapeutic effects observed were primarily due to improved drug delivery rather than glial activation or immune-mediated mechanisms related to FUS-induced stress.2

## Discussion

This study demonstrates that FUS-mediated BBBO significantly enhances TMZ delivery, improving tumor suppression and survival in an orthotopic GBM xenograft mouse model. Our findings highlight that early-stage BBB modulation before neovascularization provides a critical therapeutic window for improving drug penetration. In contrast to previous studies that focused on FUS after tumor vascularization, our results suggest that BBB permeability changes are most effective when the barrier remains intact, before tumor-induced vascular remodeling occurs (Fig. 4). These findings indicate that addressing the drug delivery limitations reported for numerous GBM treatment candidates [35–38] could create opportunities for the application of a broader range of therapeutic agents.

Although TMZ is known to penetrate the BBB because of its small molecular weight and lipophilic properties, its permeability is still significantly lower in intact BBB regions than in areas with a disrupted barrier [39, 40]. Studies have shown that while TMZ can reach brain tumors, its concentration in the cerebrospinal fluid remains as low as 20% of plasma levels, indicating that BBB integrity limits drug bioavailability [41, 42]. Given that multiple clinical studies have demonstrated the safety of repeated FUS-mediated BBBO, enhancing BBB permeability through FUS remains a promising strategy for improving TMZ delivery to infiltrative GBM cells in regions with an intact BBB [43].

A key finding of this study is that FUS significantly increases intratumoral TMZ concentrations, as demonstrated quantitatively via LC-MS/MS (Fig. 5C). This enhanced TMZ penetration was closely correlated with significantly reduced tumor progression, as shown by bioluminescence imaging (Fig. 5A, B). Additionally, Kaplan–Meier survival analysis further supported these findings, demonstrating that FUS + TMZ significantly prolonged survival compared with TMZ alone (Fig. 5), consistent with previous studies demonstrating FUS-enhanced chemotherapy efficacy [44]. These results suggest that enhanced BBB permeability via FUS can improve the therapeutic efficacy of TMZ and also various currently identified GBM treatment candidates [27, 31, 45].

The optimal timing for FUS administration can be considered in two key aspects: (1) early treatment before tumor neovascularization (pre-vascularization phase) and (2) the timing of FUS application during the TMZ administration period (first, third, and fifth days of treatment).

Regarding early treatment timing (pre-vascularization), FUS may be therapeutically advantageous when applied while the BBB remains intact, as this could facilitate a more uniform distribution of TMZ. In our study, MRI and BBB permeability assay confirmed that the BBB remained intact in the tumor-only group, suggesting that neovascularization had not yet developed at the time of treatment (Fig. 4A–F). Given that all TMZ-related groups received the same dosing schedule, the observed differences in therapeutic outcome likely reflect variations in drug delivery efficiency associated with the timing of FUS application, rather than differences in BBBO or TMZ exposure itself. This finding supports the hypothesis that BBBO via FUS in the early-stage tumor environment can enhance TMZ delivery before the formation of abnormal vasculature complicates drug penetration.

In addition to pre-vascularization treatment, the timing of FUS during the TMZ administration cycle is another critical factor. To determine the optimal timing, FUS was applied on the first, third, and fifth days of TMZ treatment. As shown in Supplementary Fig. 1, FUS treatment generally exhibited a trend toward prolonged survival. Notably, compared with the Control and TMZ-only groups, only the 1-day FUS group demonstrated a statistically significant delay in tumor progression and extended survival. This suggests that early BBB modulation through FUS may enhance TMZ distribution and therapeutic efficacy, although further validation is required [6, 46].

Previous studies have explored different FUS administration schedules, with some suggesting that repeated or later-stage treatments may enhance drug uptake as tumor vascularization increases [24, 44]. However, as the tumor progresses, hypoxia-driven angiogenesis leads to BBB heterogeneity and BTB formation, creating a dysfunctional barrier that may limit drug penetration despite FUS treatment [47, 48]. In contrast, early-stage FUS application, where the BBB remains intact and the microvasculature is immature, could facilitate more effective TMZ delivery.

Previously, clinical trials investigating FUS for GBM treatment primarily focused on its application as salvage therapy in recurrent tumors [49]. However, by the time recurrence occurs, GBM cells often exhibit resistance to TMZ, significantly diminishing therapeutic efficacy [50]. To address this limitation, current clinical research, including an ongoing trial (NCT04614493), is evaluating the integration of FUS and TMZ during initial treatment phases in patients with newly diagnosed GBM. Our results support this approach, suggesting that early application of FUS, specifically targeting peritumoral regions post-surgery, may enhance TMZ penetration and eliminate infiltrative tumor cells before recurrence [24, 34].

A key translational challenge in GBM treatment is the early-stage monitoring of infiltrative tumor cells before vascularization. Most imaging modalities, including contrast-enhanced MRI, primarily detect tumors based on neovascularization and BBB breakdown [51]. However, early GBM infiltration into normal brain tissue often occurs without clear contrast enhancement, making it difficult to detect and treat these infiltrative cells in clinical settings [52]. This underscores the potential of FUS-mediated BBB modulation as an adjunctive therapy before tumor neovascularization, maximizing drug delivery to infiltrative tumor regions while the BBB remains intact.

A potential clinical application of our findings is the implementation of FUS-TMZ therapy in the postoperative setting, specifically targeting the peritumoral region following maximal safe resection. Given that most GBM recurrences occur near the surgical margin [53], applying FUS at this early stage could improve TMZ penetration and enhance local tumor control. Notably, the TS15-88 cell line used in this study exhibited a highly infiltrative phenotype, characterized by diffuse invasion into the surrounding brain parenchyma, which is evident only by histological evaluation using human-specific ZEB-1 immunofluorescence staining (Fig. 3). This invasive behavior closely mimics clinical settings of residual microscopic disease following surgical resection, thereby reinforcing the clinical relevance and translational potential of our model for evaluating FUS-mediated drug delivery strategies.

Although this study demonstrates the potential of FUS-enhanced TMZ delivery, several limitations must be considered. First, this study did not directly investigate TMZ resistance mechanisms, such as MGMT expression, DNA repair pathways, or tumor heterogeneity, which are critical factors influencing TMZ effectiveness. Future studies should explore whether FUS-mediated BBBO affects these resistance pathways.

Second, our model utilized only male athymic nude mice to minimize experimental variability, precluding the assessment of immune modulation, an increasingly important factor in GBM treatment. Future studies employing immunocompetent animal models and including both sexes would further elucidate whether FUS enhances TMZ efficacy through immune-mediated mechanisms, thereby strengthening clinical relevance [54, 55].

Another translational challenge is the early detection of infiltrative tumor cells. Current imaging modalities primarily detect tumors after the occurrence of neovascularization and contrast enhancement. Incorporating advanced imaging techniques such as dynamic contrast-enhanced MRI or molecular biomarkers in future research may help identify the optimal timing for early-stage FUS intervention. Additionally, in our current study, treatment was initiated at an early stage (1 week after tumor implantation), potentially raising concerns about treatment effects on tumor engraftment rather than tumor growth. However, our immunofluorescence analyses using GFAP and Iba-1 (Supplementary Fig. 2) confirmed robust glial and microglial activation in tumor-bearing groups, strongly indicating successful tumor engraftment. Further histological evidence, such as human-specific ZEB-1 staining (Fig. 3), also confirmed effective tumor implantation at this early time point. Nevertheless, future studies incorporating additional PDX or cell-line models with treatments initiated at more clearly visible, advanced tumor-engraftment stages will be valuable for further validating our findings and enhancing their clinical relevance.

Future research should investigate the comparative benefits of early versus late FUS application, clarify the potential advantages of repeated multiple FUS cycles, since our study evaluated only a single treatment cycle, and explore how different tumor vascularization stages influence drug delivery efficiency. Clarifying these factors is crucial for refining patient-specific FUS-TMZ treatment strategies. Despite these limitations, our findings provide compelling preclinical evidence supporting FUS as a promising approach to enhance TMZ efficacy, particularly when applied before tumor neovascularization.

## Conclusion

This study demonstrates that FUS-mediated BBBO enhances TMZ delivery, reduces tumor burden, and prolongs survival in an orthotopic GBM xenograft model. Notably, early FUS administration exhibited a trend toward improved tumor suppression and survival, suggesting its potential therapeutic advantage in optimizing chemotherapy delivery. Given that most GBM recurrences occur near the resection margin, integrating FUS with standard concurrent chemoradiotherapy and adjuvant TMZ, particularly through wide-field application targeting the MR-visible tumor periphery, could enhance drug penetration into infiltrative tumor regions, lower recurrence rates, and ultimately improve survival outcomes. Future studies incorporating vascular imaging, immune profiling, and resistance analysis are required to determine the full impact of FUS timing and spatial coverage on GBM treatment efficacy. Ultimately, this study highlights the potential clinical relevance of FUS as an adjunct therapy to standard GBM treatment, particularly when applied in the early stage post-surgical setting to enhance chemotherapy distribution and improve long-term patient outcomes.

## Supplementary Information

Below is the link to the electronic supplementary material.

Supplementary Material 1

## Data Availability

The data that support the findings of this study are available from the corresponding author.

## Abbreviations

ANOVA: Analysis of variance
BBB: Blood–brain barrier
BBBO: Blood–brain barrier opening
BTB: Blood–tumor barrier
FUS: Focused ultrasound
GBM: Glioblastoma
GFAP: Glial fibrillary acidic protein
H&E: Hematoxylin and eosin
MBP: Myelin basic protein
MRI: Magnetic resonance imaging
NeuN: Neuronal nuclei
TMZ: Temozolomide
TS: Tumorsphere
TUBB3: Tubulin beta 3
