# Supplementary material for: Focused ultrasound-mediated temozolomide delivery into intact blood–brain barrier tissue improves survival in patient-derived xenograft model of glioblastoma
Source: Fluids Barriers CNS. 2025 Aug 25;22:87. doi: 10.1186/s12987-025-00695-0 (PMC12376472; doi:10.1186/s12987-025-00695-0)
Supplement: Supplementary file 1 — Supplementary Material 1 [file 12987_2025_695_MOESM1_ESM.docx]

**Supplementary figures**

**
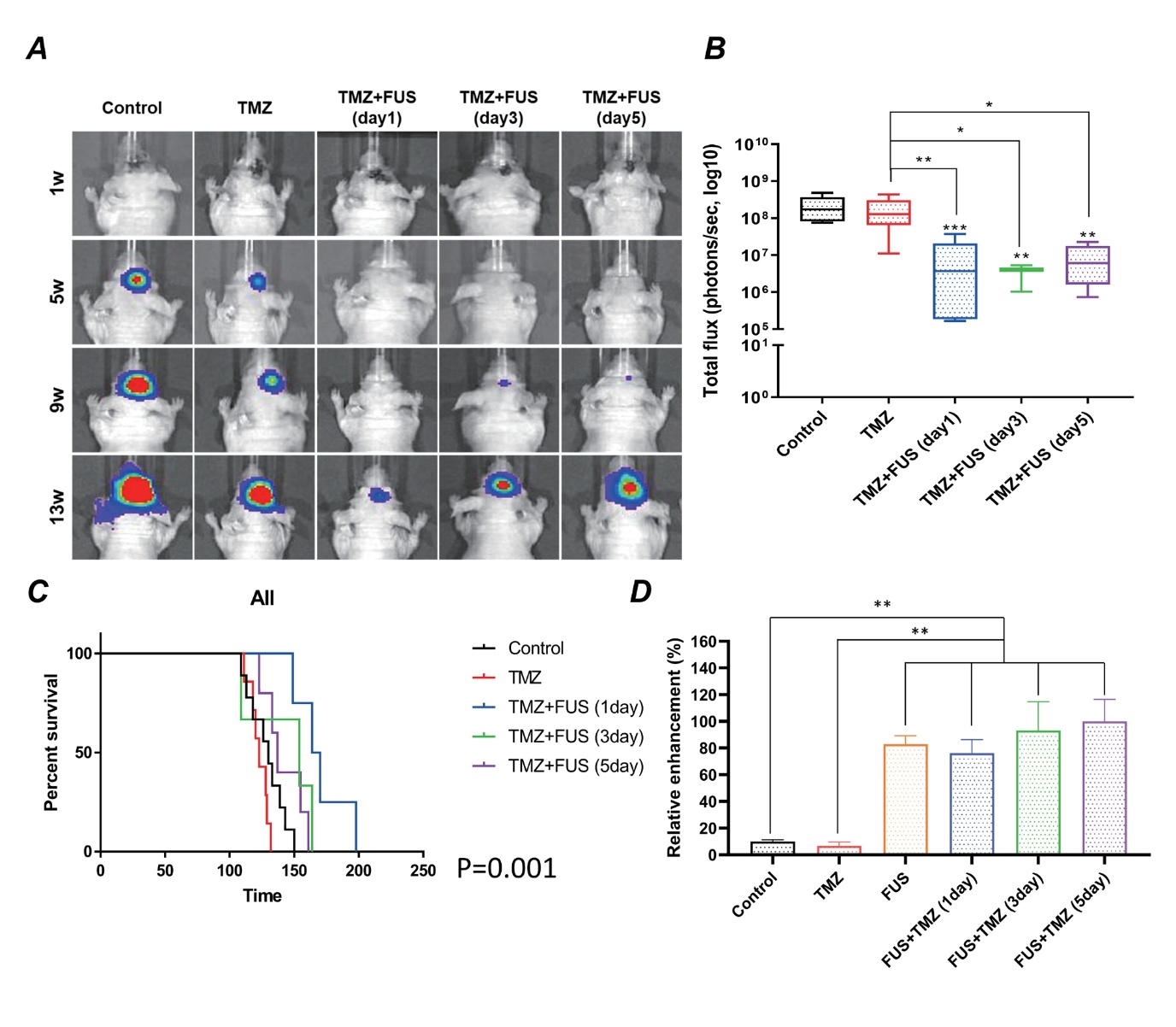
**

**Supplementary Figure 1** Evaluation of FUS timing on tumor progression and survival. **(A)** Bioluminescence imaging of tumor growth in mice subjected to FUS at different time points (1 day, 3 days, and 5 days post-xenograft implantation). Representative images are shown for weeks 1, 5, 9, and 13. **(B)** Quantification of total photon flux from bioluminescence imaging, representing the tumor burden over time for each FUS timing group. **(C)** Kaplan–Meier survival analysis comparing the survival rates among the 1-day, 3-day, and 5-day FUS groups. The 1-day FUS group exhibited a significantly higher survival rate than the control and TMZ-only groups. However, while FUS administration at other time points showed a trend toward improved survival, the differences were not statistically significant. Statistical analysis was performed using the log-rank test. **(D)** Quantification of T1-weighted gadolinium-enhanced MRI signal changes, presented as relative enhancement. Images were acquired immediately post-FUS, and data are shown as mean ± SEM (n = 3 per group).

**
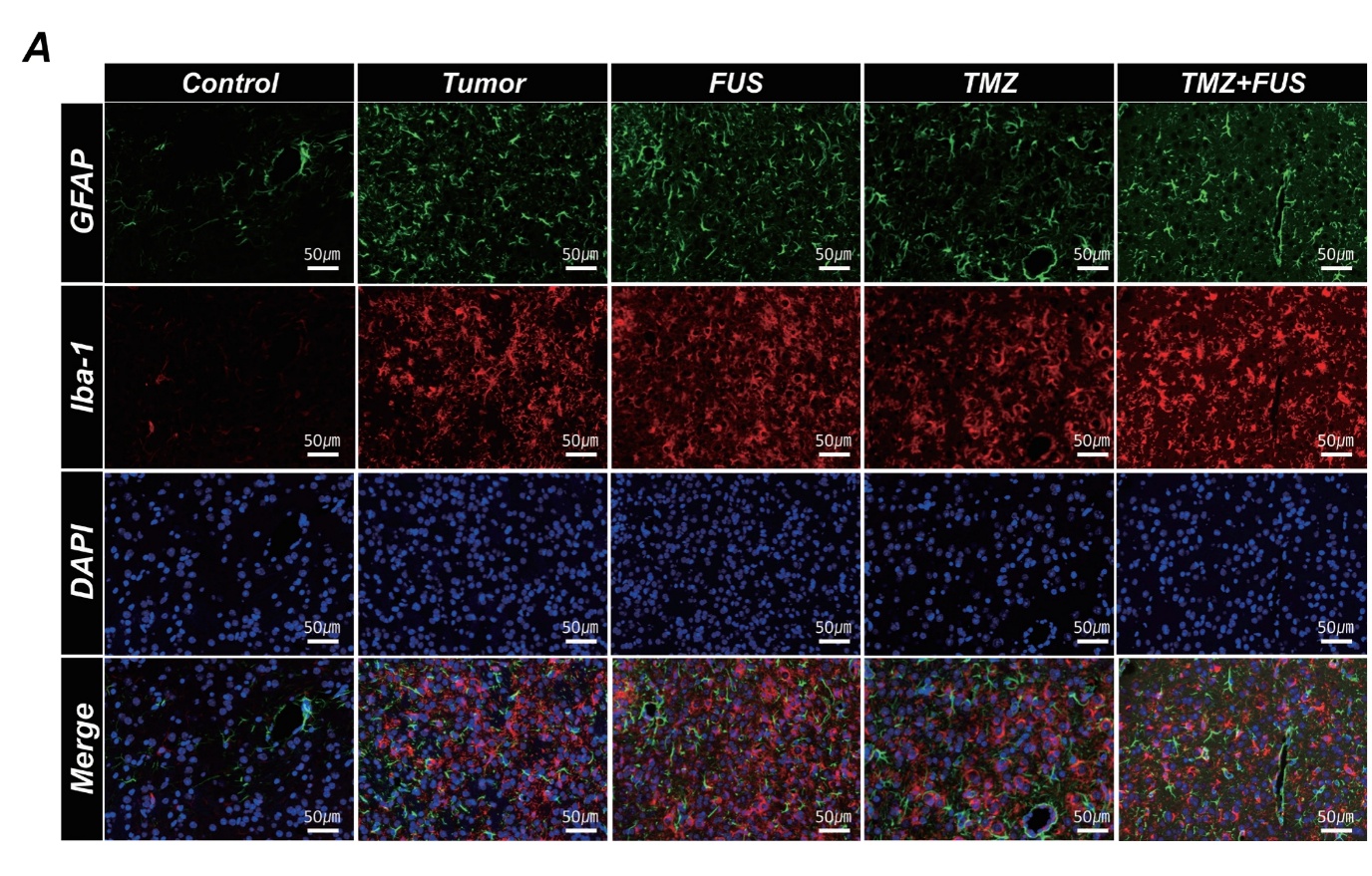
**

**Supplementary Figure 2** Evaluation of glial and immune cell activation following FUS and TMZ treatment. **(A)** Representative immunofluorescence staining for microglial activation (Iba-1, green), astrocytic activation (GFAP, red), and nuclear staining (DAPI, blue) in Control, Tumor-only, FUS-only, TMZ-only, and TMZ+FUS groups. No significant differences were observed in Iba-1 and GFAP expression among all groups, indicating that the therapeutic effects observed were primarily due to enhanced TMZ delivery rather than immune modulation or glial activation induced by FUS treatment. Scale bars: 50 µm.
